# Novel Polymeric Thin-Film Composite Membranes for High-Temperature Gas Separations

**DOI:** 10.3390/membranes9040051

**Published:** 2019-04-10

**Authors:** Fynn Weigelt, Sara Escorihuela, Alberto Descalzo, Alberto Tena, Sonia Escolástico, Sergey Shishatskiy, Jose Manuel Serra, Torsten Brinkmann

**Affiliations:** 1 Helmholtz-Zentrum Geesthacht, Institute of Polymer Research, Max-Planck-Str.1, 21502 Geesthacht, Germany; fynn.weigelt@hzg.de (F.W.); saesro@itq.upv.es (S.E.); albertodescalzo5@gmail.com (A.D.); sergey.shishatskiy@hzg.de (S.S.); 2 Instituto de Tecnología Química, Universitat Politècnica de València-Consejo Superior de Investigaciones, Científicas, Avda. Los Naranjos, s/n, 46022 Valencia, Spain; soesro@upvnet.upv.es

**Keywords:** thin-film composite membranes, high-temperature applications, high thermal stability, hydrogen, carbon dioxide

## Abstract

Novel selective polymeric thin-film composite membranes (TFCMs) for applications at elevated temperatures were developed. Thin selective layers of the polyimides Matrimid 5218^®^ and 6FDA-6FpDA were cast on a developed polybenzimidazole (PBI) porous support prepared by a phase inversion process. The TFCM properties were investigated with different gases in a wide temperature range, including temperatures up to 270 °C. The membranes showed very high thermal stability and performed well at the elevated temperatures. The development of highly thermally resistant polymeric membranes such as these TFCMs opens opportunities for application in high-temperature integrated processes, such as catalytic membrane reactors for the water-gas shift reaction in order to maximize H_2_ yield.

## 1. Introduction

Membranes offer attractive opportunities for a great number of gas separation applications, such as air separation, hydrogen purification, or natural gas upgrading [1]. Considering the nature of the membrane selective layer, membranes can be classified as polymeric, metallic, ceramic, and carbonaceous [1,2,3]. Materials are selected according to the application conditions and requirements. Although polymeric membranes exhibit clear benefits in terms of processability and relatively low price, they also have some drawbacks, such as low thermal and chemical stability, that have to be carefully considered with respect to the selected application [4]. 

In order to apply the selective materials in an industrial process, the polymeric membranes must be processed as a thin selective layer (<100 nm). Since the layer is very thin, the materials must be supported [5,6,7]. One method to obtain a supported selective layer is by the formation of thin-film composite membranes (TFCMs). In this case, the supporting structures should possess good mechanical properties and, together with the selective layer, should have high thermal and chemical stability, whilst simultaneously being highly permeable. Obviously, a thinner selective layer leads to higher flux through the membrane. The multilayer concept of TFCMs is very attractive in terms of simplicity and cost compared to integral asymmetric membranes, where the material of the selective layer needs to be the same as the material of the porous support. This fact opens the way for practical use of expensive tailor-made polymers and other materials which are often too expensive or not stable enough to be used for the formation of the whole membrane structure [8]. There are multiple materials with promising properties for specific applications but with applicability limitations such as in cost, upscaling, stability, or mechanical or other physical-chemical properties. These problems can be solved by using inexpensive materials as porous support, and by placing a very thin layer of the selective—and more expensive—material on top [6,7]. In this way, we can match the selective layer and the support material for a specific application. 

Typical polymeric supporting materials are made from commercial materials such as polyacrylonitrile or polyetherimide [6,9,10]. These porous support materials are not stable at elevated temperatures (greater than 180–200 °C). This issue can be solved by using more thermally stable supports, such as ceramics. The main limitation of this approach is the compatibility between ceramic support and the polymeric layer. This problem was solved by studying the limitations of the coating process [11]. Despite the high thermal and chemical stability, the use of ceramic supports presents some limitations [12], especially in terms of compatibility with the polymeric layer. In terms of the selective layer, glassy polyimides present excellent separation properties as well as very good thermal and chemical stability [13,14,15,16]. Therefore, the ideal situation can be found when both support and selective layer are polymeric materials.

In polymeric gas separation membranes, the mass transfer is described according to the solution–diffusion mechanism [1]. In the solution–diffusion theory of molecular transport in polymers, the permeability (*P_i_*) coefficient is determined by two factors (Equation (1)): The solubility, *S_i_*, related to the properties of the gas and to its interaction with the polymer matrix. It reflects the number of molecules dissolved in the membrane material. Since it depends on molecular interaction, it is an equilibrium term. On the other hand, the diffusivity, *D_i_*, depends mainly on the ability of the gas molecules to move through the bulk of a polymer by migrating from one free volume void to another.

(1) $$P_{i}=D_{i}\cdot S_{i}$$

At elevated temperatures, as in the case of several industrial processes, the solubility factor becomes less important compared to the more dominant diffusivity factor compared to ambient temperatures. Therefore, under the here-described conditions, the major influence of the permeability selectivity (Equation (2)) is mostly seen in the differences in the diffusion coefficients of gases dissolved in the polymer. An additional explanation employs the temperature dependencies of the two coefficients. The heat of sorption Δ*H_S_* consists of the heat of mixing and the heat of condensation, and can be either positive or negative [17]. The activation energy of diffusion *E_d_* is always positive, and for permanent gases its absolute value always is considerably larger than the absolute value of the heat of sorption [18].

(2) $$\alpha_{ij}=\alpha_{D}\cdot \alpha_{S}=[\frac{D_{i}}{D_{j}}]\cdot \left[\frac{S_{i}}{S_{j}}\right]$$

Especially for polymers, this consideration limits the choice of selective materials to glassy polymers, where permeability selectivity is directed by the diffusion selectivity, and not by the solubility selectivity. The largest use of syngas is hydrogen production, where steam methane reforming (SMR) is the predominant technology. The latter is a well-established process with two main reactions: reforming and water-gas shift (WGS) reactions [12,19,20]. 

The endothermic methane steam reforming reaction is:

(3) $${\mathrm{CH}}_{4}+H_{2}O⇌\;\mathrm{CO}+3H_{2}\;\;\;\Delta H=+206.2\;\frac{\mathrm{kJ}}{\mathrm{mol}}.$$

Afterwards, the carbon monoxide reacts further with steam to form H_2_ and CO_2_, by the weakly exothermic water-gas shift reaction:

(4) $$\mathrm{CO}+H_{2}O⇌{\;\mathrm{CO}}_{2}+H_{2}\;\;\;\;\Delta H=-41.2\;\frac{\mathrm{kJ}}{\mathrm{mol}}\;$$

In order to use this hydrogen, a purification step of the mixture CO, CO_2_, and H_2_ is necessary. The composition of the mixture varies depending on the source and the intended use of the synthesis gas. A typical example is ammonia synthesis, where the gas exiting the low-temperature water-gas shift reaction contains 44% hydrogen, 13% CO_2_, and 28% water, among other gases [21]. Low-temperature WGS reactors using Cu-Zn catalyst systems are operated at about 200 °C [21]. For high-temperature WGS reactors employing Fe-CR catalysts, membranes that can withstand temperatures above 300 °C and upstream pressures of up to 2.7 MPa are needed [22].

In the literature there are only a few works available discussing high-temperature measurements of polymeric gas separation membranes. Most of them are about hollow fiber polybenzimidazole (PBI) membranes [23,24,25]. Costello and Koros describe dense films of 6FDA-6FpDA and 6FDA-6FmDA membranes at elevated temperatures up to 300 °C [26].

To expand the applicability of polymeric materials to these types of high-temperature processes, it is necessary to provide new thermally and chemically stable porous support structures to produce TFCMs. Therefore, in this work, a highly thermally resistant polymeric porous support is developed and combined with dense layers of thermally stable polymers. The characterization was performed taking into account the later application for hydrogen separation. Efforts were carried out to improve the membrane stability, enabling its operation temperature up to 270 °C. This is a temperature range which is interesting for separation processes in the petrochemical industry and for use in catalytic membrane reactors.

## 2. Experimental

### 2.1. Materials

#### 2.1.1. Non-Woven Support

The non-woven polyphenylene sulfide (PPS) has a high porosity as well as excellent thermal and chemical stabilities. The supplier cannot be disclosed due to license issues. 

#### 2.1.2. Polymers

Polybenzimidazole (PBI), a poly[2,2′-(*m*-phenylene)-5,5′-bibenimidazole], was purchased from PBI^®^ Performance products, Inc. (Charlotte, NC, USA) in a 26 wt. % solution. Polyethylene glycol (PEG) with an average molecular weight of 2000 g mol^−1^ was obtained from Sigma-Aldrich (St. Louis, MO, USA). The polyimide Matrimid^®^ 5218 (Matrimid^®^) was purchased from Huntsman Advanced Materials GmbH (Berkamen, Germany) in powder form. The polyimide 6FDA-6FpDA was synthesized for the current work according to procedure described elsewhere [27]. Teflon^®^ AF 2400 was purchased from E. I. du Pont de Nemours (Wilmington, DE, USA).

#### 2.1.3. Solvents

Tetrahydrofuran (THF), dimethyl acetamide (DMAc), and toluene were purchased from Merck GmbH (Darmstadt, Germany). 3M^TM^ Fluorinert^TM^ FC-770, >99%, was purchased from IOLITEC Ionic Liquids Technologies GmbH (Heilbronn, Germany). All solvents were used as received.

#### 2.1.4. Gases

Gases were purchased from Linde AG and had purities of at least 99.99%.

### 2.2. Membranes

#### 2.2.1. Thick Films

Dense homogeneous films of Matrimid^®^, 6FDA-6FpDA, and Teflon^®^ AF 2400 were prepared by casting the polymer solution (in THF and FC-770 for Teflon^®^ AF 2400) on a leveled glass plate and drying under constant dry nitrogen flow. When the solvent evaporation was complete, the membranes were dried for 6 h at 200 °C under vacuum. The thickness of the homogenous membranes was determined using a Fischer Deltascope FMP10 (Helmut Fischer GmbH, Sindelfingen, Germany). The thickness of the Matrimid^®^ dense film was 30.1 ± 0.1 μm, the thickness of the 6FDA-6FpDA was 16.8 ± 0.1 μm, and the thickness of the Teflon^®^ AF 2400 was 25.4 ± 0.1 μm.

#### 2.2.2. Preparation of TFCMs

**Figure 1:**
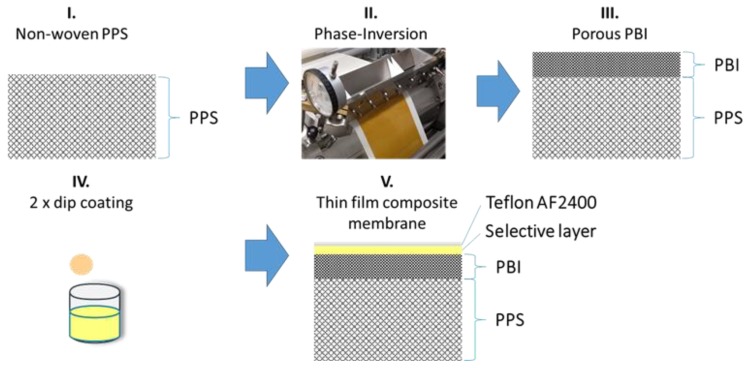
Sketch of the production steps to form thin-film composite membranes (TFCMs) with four layers: polyphenylene sulfide (PPS) as a non-woven support, polybenzimidazole (PBI) as a porous support, Matrimid^®^ or 6FDA-6FpDA as a selective layer, and Teflon^®^ AF 2400 as a protective layer.

The porous support for the TFCMs (Figure 1) were prepared on a non-woven PPS support on a lab-scale membrane casting machine [28]. First the purchased PBI solution was diluted with DMAc to the concentration of 18 wt. % of PBI. As a porosity modifier, 6 wt. % PEG 2000 was added to the solution. On the lab-scale casting machine, the PBI solution was cast on the non-woven PPS and the porous PBI support was obtained by phase inversion [29]. Water was used as a non-solvent of the coagulation bath at a temperature of 20 °C. After the casting, the porous support was extensively washed with an excess of hot water and dried afterwards.

TFCMs were coated by a dip-coating process. The porous support was first dipped in toluene in order to fill the pores. Subsequently, the samples were dip coated with the polymer solution (4 wt. % Matrimid^®^ or 3.5 wt. % 6FDA-6FpDA) and subsequently dried at 50 °C for 24 h. Afterwards, the samples were dip coated in a solution of 1 wt. % Teflon^®^ AF2400 in FC 770 to get a protective layer. Time of dip coating was controlled to 10 s. The TFCMs were dried in an oven under vacuum for 24 h at 260 °C to ensure that all the solvent used during the process was evaporated [30].

### 2.3. Characterization Methods

The “time-lag” and the “pressure increase” (variable pressure, constant volume [31]) methods for thick dense films and for TFCMs, respectively, were used to determine the gas transport parameters. The temperature ranged from 30 to 70 °C and a 100 mbar feed pressure was used for H_2_, N_2_, O_2_, CO_2_, and CH_4_. The first method relied on maintaining a constant feed pressure while monitoring the permeate pressure, which changed as a function of time due to the transport of gas molecules through the membrane. The measurement of the increase in pressure in the permeate chamber of known constant volume started at the time point when the gas at the constant pressure was brought in contact with the membrane. The time-lag, *θ*, was determined by extrapolating the slope of the linear increase to its intersection with the time axis. Additionally, the gas permeability coefficient of the membrane was calculated from the linear part of the curve. The permeability (*P_eff,i_*) of a gas penetrant *i* is the pressure or fugacity difference (i.e., driving force) and thickness-normalized flux of the component through the membrane and is defined by:

(5) $$P_{eff,i}=\;\frac{N_{i}l}{A_{m}\Delta p_{i}},$$

where *P_eff,i_* is the permeability of component *i*, *N_i_* is the volumetric flowrate of the component *i* at standard conditions (STP) through the membrane, *A_m_* is the membrane area, and Δ*p_i_* the partial pressure (or fugacity) difference between feed and permeate sides. The single gas permeance *L_eff,i_* can be calculated by:

(6) $$L_{eff,i}=\frac{P_{eff,i}}{l}=\frac{N_{i}}{A_{m}\Delta p_{i}}.$$

The volumetric flowrate was determined from the permeate pressure change with time assuming that the ideal gas law is applicable in the pressure range of 0–10 mbar (i.e., the working range of the permeate-side pressure sensor). The ideal selectivity *α* of a dense gas separation membrane is defined as:

(7) $$\alpha_{i,j}=\frac{P_{i}}{P_{j}}=\frac{L_{i}}{L_{j}},$$

where *P_i_* and *P_j_* are the permeabilities of gases *i* and *j*.

A custom-built machine was used for the “time-lag” and “pressure-increase” measurements. The machine employs two software based evaluation modes: one designed for experiments involving thick isotropic films and allowing for determination of the diffusion, solubility, and permeability coefficients of gases; and the second designed for the determination of the gas transport properties of practical membranes having selective layers thin enough to neglect the time lag caused by the diffusion of gas molecules through the bulk of the selective layer material.

**Figure 2:**
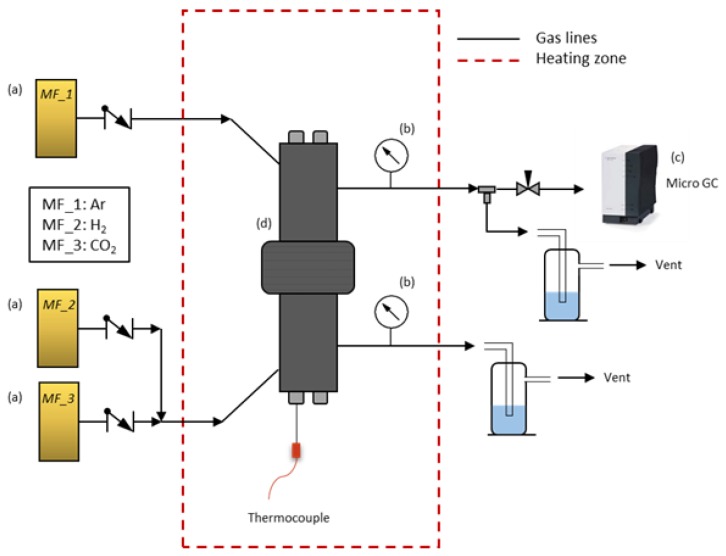
High-temperature set-up for polymeric membranes.

Single gas measurements at high temperature were performed in a module designed for this purpose. The set-up (Figure 2) consisted of: (a) three mass flow controllers (MF_1–MF_3) for H_2_ and CO_2_ as feed gases and Ar as sweep gas; (b) two manometers to control the internal pressure in both chambers of the reactor (feed side and permeate side); (c) a micro GC to measure the outlet gases at the permeate side; and finally (d) a reactor with two chambers that allow the membrane to be placed and sample to be sealed by rubber rings in the center. Membranes were placed in the center of the reactor and sealed from both sides. Hence, two well-defined chambers separated by the membrane sample allowed us to perform measurements at different temperatures.

Fluxes (*J_i_*), in mL_STP_ m^−2^ s^−1^, of H_2_ and CO_2_ permeating through the membrane were calculated by dividing the gas concentration by the effective surface area of the membranes *A_m_*, as shown in the following equation:

(8) $$J_{i}=\frac{{\%}_{i}\cdot Q_{sweep}}{A_{m}}.$$

Afterwards, flux was divided by the gas partial pressure difference (∆*p_i_*) between the two chambers, and the permeance of the specific gas could be obtained. In order to compare the permeances, the units were converted to m_STP_^3^ m^−2^ h^−1^ bar^−1^.

(9) $$P_{i}=\frac{J_{i}}{\Delta p_{i}}$$

Differential scanning calorimetry (DSC) experiments were carried out using the calorimeter DSC 1 (Mettler-Toledo, Gießen, Germany) with the following parameters: nitrogen atmosphere, heating rate 10 K/min, temperature range from room temperature to 380 °C. Three heating-cooling cycles were conducted with a five-minute isotherm interval between the heating and the cooling, whereas the first heating interval served to erase the sample history from the sample preparation (start temperature: RT, maximal temperature: 180 °C), while the other two cycles were applied for the determination of thermal properties. The latter cycles were accomplished in the temperature range 200–380 °C. In this setting, the second heating interval was used for the evaluation of the glass transition and the third heating interval was used as verification. The glass transition temperature *T_g_* was considered as the inflection point of the heat flow as a function of the temperature with the onset method using the instrumentation software. The midpoints were also estimated using this software. For the measurement, approximately 10 mg of the vacuum dried polymer and the ground composites were placed in an aluminum pan of 10 μL.

Thermogravimetric analysis (TGA) was carried out using a TG 209 F1 Iris (Netzsch, Selb, Germany). The experimental setup was as follows: temperature range 30–800 °C, heating rate 10 K/min, nitrogen atmosphere.

The morphology of the membranes was determined by scanning electron microscope (Merlin and Auriga; Carl Zeiss GmbH, Oberkochen, Germany). For this analysis the samples were either cryo-fractured in liquid nitrogen or cut with a focused gallium ion beam (FIB). Afterwards, the samples were sputter-coated with carbon.

## 3. Results and Discussion

The glass transition temperature (*T_g_*) was determined by DSC. The *T_g_* of Matrimid^®^ is 320 °C and the *T_g_* of 6FDA-6FpDA is 310 °C, as reported in previous works [11,32]. The *T_g_* of the PBI was 427 °C. These values clearly indicate the applicability of the chosen materials for application in the desired temperature range above 200 °C.

**Figure 3:**
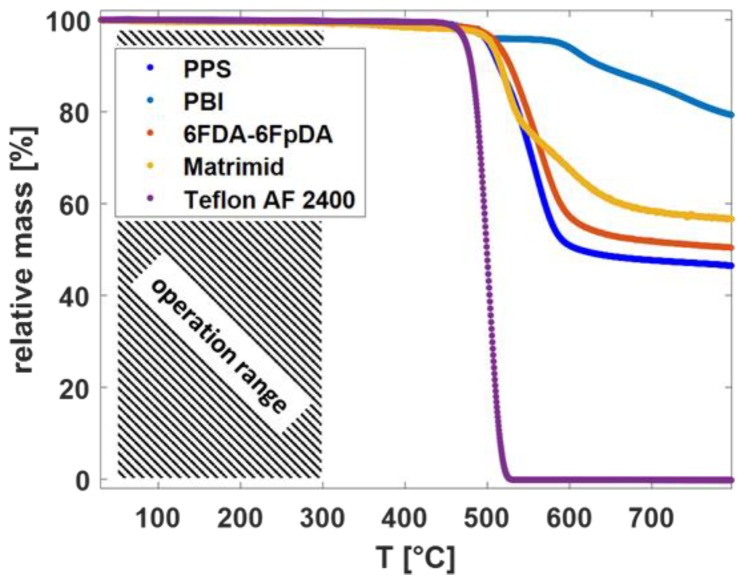
TGA thermographs for the different polymeric materials in dry Ar.

From the TGA curves in Figure 3, it is observed that the different polymeric materials showed high thermal stability. The decomposition started above 300 °C for each polymer.

**Figure 4:**
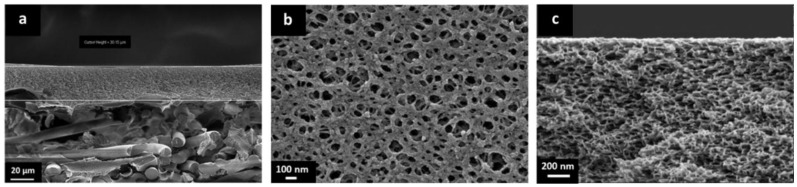
(**a**) SEM cross section of the support of non-woven PPS and porous PBI structure; (**b**) SEM surface of the porous PBI structure; and (**c**) SEM cross section of the porous PBI structure.

**Figure 5:**
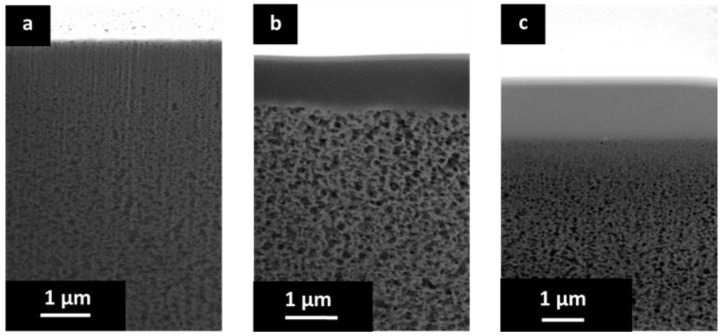
(**a**) SEM cross section of porous PBI structure; (**b**) SEM cross-section of the TFCM with a selective layer of Matrimid^®^ and Teflon^®^ AF 2400 protective layer; and (**c**) SEM cross section of the TFCM with a selective layer of 6FDA-6FpDA and Teflon^®^ AF 2400 protective layer.

The cross-sectional morphology of the porous PBI and the prepared TFCMs was analyzed. The cross section of the porous support is presented in Figure 4 and Figure 5. In the lower part of Figure 4 the non-woven PPS fibers and the porous PBI layer on top can be seen. The porous PBI layer had a thickness of approximately l ≈ 30 μm, average pore size of about 100 nm, and a surface porosity of approximately 21%. The nitrogen permeance of the porous PBI was 459 m_STP_^3^ m^−2^ h^−1^ bar^−1^, and the permeances of the other gases were in agreement with Knudsen-type gas flow through porous media.

Figure 5a shows a higher magnification for the cross section of the porous PBI. In this case, the sample was cut by FIB. The anisotropic nature of the porous PBI structure can be clearly seen, as can pores on the membrane surface.

Figure 5b shows the cross-sectional morphology for the TFCM with a Matrimid^®^ selective layer. The Matrimid^®^ layer showed a thickness of about 1 μm while the thickness of the Teflon^®^ AF 2400 protective layer was estimated to be under 80 nm. The selective layer and the protective layers were homogenous, without visible defects. No penetration of the Matrimid^®^ into the pores was observed. Figure 4c shows the cross-sectional morphology of the TFCM with 6FDA-6FpDA as the selective layer. According to Figure 5c, the selective layer of 6FDA-6FpDA had a thickness of about 1 μm and the protective layer on top of Teflon^®^ AF 2400 was estimated to have a thickness of 100 nm. Figure 5c shows evidence of 6FDA-6FpDA penetration into the pores of the PBI substrate, which was filled with toluene to prevent polymer penetration during the membrane preparation. In this case, the polyimide could withstand the presence of toluene in the polymer/THF solution without going into phase inversion. Therefore, the presence of toluene in the PBI structure resulted not just in partial polyimide penetration into the pores but also in toluene penetration into the coating solution as well, causing partial phase inversion in the coating polymer solution during the selective layer drying. This could explain the difference in gas transport results for the TFCM and isotropic 6FDA-6FpDA film discussed below, as well as the differences in the selective layer thickness estimated from SEM images and from gas transport measurements. This was not the case for the Matrimid^®^, where toluene did not allow the polymer to penetrate into the PBI porous structure and, therefore, the selective layer thickness estimated from SEM and gas transport analysis were in good agreement.

The time-lag method was used for the evaluation of the permeability coefficients of the Matrimid^®^ and 6FDA-6FpDA thick films for H_2_, CO_2_, O_2_, CH_4_, and N_2_ in the temperature range 30–70 °C, at 1000 mbar feed pressure (Table 1). The ideal gas perm-selectivities at 30 °C were *α*_H2/CO2_ = 2.6, *α*_O2/N2_ = 6.2, and *α*_CO2/CH4_ = 35.7 for Matrimid^®^ and *α*_H2/CO2_ = 1.4, *α*_O2/N2_ = 5.4, and *α*_CO2/CH4_ = 47.6 for 6FDA-6FpDA. The activation energy (*E_A,i_*) of permeability was calculated from data obtained in the range 30–70 °C and is defined from the Arrhenius equation as:

(10) $$P_{i}\left(T\right)=P_{0,i}\cdot e^{\frac{-E_{A,i}}{R\cdot T}},$$

where *P_i_* is the permeability, *P*_0*,i*_ is the pre-exponential factor, *R* is the universal gas constant, and *T* is the temperature.

The pressure increase method was used for the H_2,_ CO_2_, O_2_, CH_4_, and N_2_ permeance evaluation of the TFCMs with selective layers of Matrimid^®^ and 6FDA-6FpDA and the Teflon AF 2400 protective layer. As in the case of the thick films, the pure gas permeance data was acquired for the TFCMs in the temperature range 30–70 °C and the data for 30 °C are presented in Table 2. The selectivities in the case of the Matrimid^®^ membrane were *α*_H2/CO2_ = 2.2, *α*_O2/N2_ = 6.4, and *α*_CO2/CH4_ = 35.2; for 6FDA-6FpDA they were *α*_H2/CO2_ = 1.1, *α*_O2/N2_ = 4.1, and *α*_CO2/CH4_ = 33.6 at 30 °C. The Matrimid^®^ TFCMs selectivities agree with the selectivities of the thick film membranes, whilst for the 6FDA-6FpDA TFCMs the selectivities were lower than the ideal selectivities calculated from permeability coefficients. The same trend was observed in the activation energies of the 6FDA-6FpDA membranes. The *E_A_* values of the Matrimid^®^ TFCMs were in good agreement with the *E_A_* values of the thick-film membranes. For 6FDA-6FpDA, a decrease of the activation energies in the TFCMs was observed. A possible explanation could be the influence of the Teflon^®^ AF 2400 protective layer on overall membrane performance. Table 1 displays the permeabilities and *E_A_* values of the Teflon AF 2400. Teflon AF 2400 had high permeabilities and low selectivities. The selectivities for Teflon^®^ AF 2400 were *α*_H2/CO2_ = 1.0, *α*_O2/N2_ = 2.3, and *α*_CO2/CH4_ = 8.1 at 30 °C. In Table 2, the estimated selective layer thicknesses are presented, as calculated from the gas transport data obtained for isotropic polymer films and TFCMs (i.e., by dividing the permeability by the permeance). For Matrimid^®^ TFCM the calculated selective layer thickness was in agreement with the estimated thickness from the SEM image (Figure 5). For 6FDA-6FpDA the calculated selective layer thickness was lower than the estimated layer thickness from the SEM image (Figure 5c). This could explain the difference in the *E_A_* values for the TFCM of 6FDA-6FpDA, which may have originated from the observed penetration and partial phase inversion of the 6FDA-6FpDA in Figure 5c. Therefore, the protective Teflon^®^ AF 2400 layer appeared to have a significant influence on the permeance of the 6FDA-6FpDA TFCM. The Teflon^®^ AF 2400 was selected based on its high permissibility to the transport of the gases and due to its high thermal stability. From this, it can be inferred that a much thinner layer of the protective fluorinated polymer should be applied for a fast polymer such as 6FDA-6FpDA.

**Figure 6:**
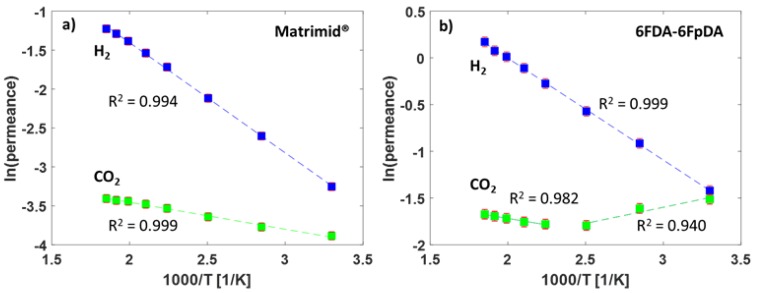
(**a**) Arrhenius plot for the TFCMs of Matrimid^®^ and (**b**) 6FDA-6FpDA for temperatures between 30 and 266 °C.

The high-temperature gas transport measurement method was used for the evaluation of the permeance of the TFCMs with the selective layers of Matrimid^®^ and 6FDA-6FpDA for the gases H_2_ and CO_2_. The pure gas permeance data were obtained for the two TFCMs in the temperature range 30–270 °C at 1 bar feed pressure and sweep gas flowrate 50 mL min^−1^. The permeance and selectivity values are presented in Table 3. It was possible to conduct reproducible gas transport measurements for the TFCMs up to 275 °C, which is in accordance with the TGA measurements (Figure 3). The temperature limit was imposed by the PPS non-woven support, as it started to melt at this point, causing the membrane to break. The H_2_/CO_2_ selectivity for Matrimid^®^ was *α*_H2/CO2_ = 1.9 and for 6FDA-6FpDA it was *α*_H2/CO2_ = 1.1 at 30 °C, which are in good agreement with the data of pressure increase measurements. Since the activation energy for CO_2_ (*E_A,_*_CO2_) is lower than *E_A,_*_H2_, the H_2_/CO_2_ selectivities increased with the temperature for both membranes. For the 6FDA-6FpDA, a change in the *E_A_* values for CO_2_ was observed. The evolution of the permeance with the temperature for the gases H_2_ and CO_2_ is shown in Figure 6. In the case of Matrimid^®^ (Figure 6a), a simple Arrhenius behavior was observed for both gases. Figure 6b shows the evolution of the permeance with reciprocal temperature for the 6FDA-6FpDA. H_2_ permeance followed a simple Arrhenius behavior in the range from 30 to 270 °C, while CO_2_ permeance exhibited a change in apparent *E_A_* values for temperatures above 170 °C for CO_2_. The absolute change of the gas permeance of CO_2_ in the 6FDA-6FpDA TFCM was very small, from 0.22 m_STP_^3^ m^−2^ h^−1^ bar^−1^ at 30 °C to 0.19 m_STP_^3^ m^−2^ h^−1^ bar^−1^ at 266 °C, while the corresponding *E_A_* in a thick film of 6FDA-6FpDA (Table 1) was nearly 0 kJ mol^−1^. Therefore, further experiments will be done to analyze this observation.

**Figure 7:**
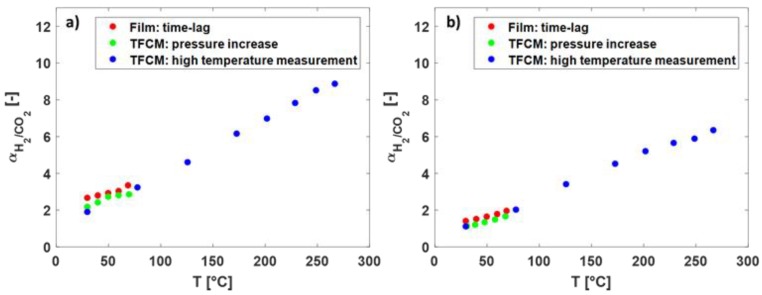
(**a**) The selectivity of H_2_/CO_2_ (-) over the temperature (°C) in Matrimid^®^ and (**b**) in 6FDA-6FpDA for different membranes and measurement methods.

In Figure 7 the selectivity of H_2_/CO_2_ over the temperature for the Matrimid^®^ and the 6FDA-6FpDA TFCM are presented. With increasing temperature, H_2_/CO_2_ selectivity increased. The selectivity of the thick-film membranes was the highest in both polymers. Between 30 and 80 °C, the selectivity followed the same trend for all membranes.

## 4. Conclusions and Outlook

A new highly thermally and chemically stable multilayer system for gas separation applications at elevated temperatures was introduced. The system was composed of four different layers: non-woven support, polyphenylene sulfide (PPS); polybenzimidazole (PBI) as polymeric porous support structure; two different polyimides—Matrimid^®^ or 6FDA-6FpDA—as selective layers; and Teflon^®^ AF 2400 as an external protective layer. The selective layers were successfully deposited as thin layers (<1 μm) on top of the porous PBI support.

These thin-film composite membrane (TFCM) systems showed high thermal stability and promising results for the separation H_2_/CO_2_ at elevated temperatures. The separation properties of the materials characterized in different thicknesses and by several techniques were compared. This multilayer approach showed thermal stability for temperatures up to 270 °C.

This type of highly thermally resistant polymeric membranes opens opportunities for application in integrated processes operated at elevated temperatures, such as membrane reactors for the water-gas shift reaction in order to maximize H_2_ yield.

Nevertheless, more efforts should be made toward the optimization of the thickness of the selective and protective layers; the stability of the non-woven support (which in this case was an important limitation); and new thermally resistant and highly selective polymers need to be tested under these conditions. Furthermore, the multicomponent permeation performance of this new type of TFCM needs to be further investigated.

## Figures and Tables

**Table 1:** Separation properties for the thick films at 30 °C and the activation energy calculated in the temperature range 30–70 °C.

Polymer	Parameter	Gases					Selectivity (-)	
		H_2_	CO_2_	O_2_	CH_4_	N_2_	O_2_/N_2_	CO_2_/CH_4_
Matrimid^®^	Permeability (Barrer) *	18.9	7.14	1.62	0.20	0.26	6.2	35.7
	*E_A_* (kJ mol^−1^)	13.0	8.15	8.93	18.0	22.6	-	-
6FDA-6FpDA	Permeability (Barrer) *	74.3	53.4	10.3	1.12	1.89	5.4	47.6
	*E_A_* (kJ mol^−1^)	3.67	0.57	3.31	7.02	6.43	-	-
Teflon^®^ AF 2400	Permeability (Barrer) *	1180	1248	504	155	222	2.3	8.1
	*E_A_* (kJ mol^−1^)	5.14	−3.01	3.40	9.12	7.38	-	-

* 1 Barrer = 10^−10^ cm_STP_^3^ cm^−1^ s^−1^ cmHg^−1^.

**Table 2:** Separation properties for the thin-film composite membranes at 30 °C and the activation energy calculated in the temperature range from 30 to 70 °C.

Polymer	Parameter	Gases				
		H_2_	CO_2_	O_2_	CH_4_	N_2_
Matrimid^®^	Permeance, 10^2^ (m_STP_^3^ m ^2^ h^−1^ bar^−1^) *	4.57	2.11	0.51	0.06	0.08
	Activation Energy (*E_A_*) (kJ mol^−1^)	12.3	6.24	7.13	20.0	14.6
	Selective layer thickness ** (nm)	1120	930	870	1280	1330
6FDA-6FpDA	Permeance, 10^2^ (m_STP_^3^ m^−2^ h^−1^ bar^−1^) *	46.1	41.3	8.35	1.23	2.05
	Activation Energy (*E_A_*) (kJ mol^−1^)	5.58	−3.45	3.08	8.95	6.51
	Selective layer thickness ** (nm)	440	350	340	250	250

* 1 m_STP_^3^ m^−2^ h^−1^ bar^−1^ = 1.239 × 10^−10^ mol m^−2^ s^−1 ^Pa^−1^; 1000 GPU = 2.700 m_STP_^3^ m^−2^ h^−1^ bar^−1^. ** The thickness of the selective layer estimated from gas transport data obtained for isotropic polymer film and TFCM.

**Table 3:** Single gas separation properties and activation energies (*E_A_*) for the thin-film composite membranes in the range of temperatures between 30 and 266 °C for the gas pair CO_2_ and H_2_. The *E_A_* values were calculated between 30 and 266 °C.

	Matrimid^®^			6FDA-6FpDA		
	Permeance, 10^2^ (m_STP_^3^ m^−2^ h^−1^ bar^−1^)					
*T* (°C)	H_2_	CO_2_	*α*_H2/CO2_	H_2_	CO_2_	*α*_H2/CO2_
30	3.86	2.05	1.88	24.2	22.0	1.10
78	7.40	2.30	3.22	40.1	20.0	2.01
125	12.0	2.62	4.58	56.5	16.6	3.40
173	18.0	2.92	6.16	76.0	16.8	4.52
202	21.4	3.09	6.92	89.6	17.3	5.18
230	25.0	3.21	7.89	101	18.0	5.61
248	27.5	3.24	8.49	107	18.4	5.82
266	29.3	3.31	8.85	118	18.8	6.28
*E_A_* (kJ mol^−1^)	11.8	2.89	-	9.02	−2.91 (30–125 °C) 2.45 (173–266 °C)	-
